# Comparative Evaluation of Sonic Vibration and Heat Preheating Techniques on the Retention and Efficacy of Flowable Composite Resin Sealants: A Quasi-experimental Study

**DOI:** 10.7759/cureus.76661

**Published:** 2024-12-31

**Authors:** Sweety Singh, Ramakrishna Yeluri, Garima Yeluri, Zakiya Perveen, Deepshikha Rajput, Rupali Malik

**Affiliations:** 1 Department of Pedodontics and Preventive Dentistry, Teerthanker Mahaveer Dental College and Research Centre, Moradabad, IND; 2 Department of Pedodontics and Preventive Dentistry, Sharad Pawar Dental College and Hospital, Wardha, IND; 3 Department of Oral Medicine and Radiology, Teerthanker Mahaveer Dental College and Research Centre, Moradabad, IND; 4 Department of Public Health Dentistry, Teerthanker Mahaveer Dental College and Research Centre, Moradabad, IND

**Keywords:** flowable composite, heat, pit and fissure sealants, vibrations, viscosity

## Abstract

Introduction: Pit and fissure sealants are crucial in reducing the incidence of dental caries, particularly in the pediatric population. Despite their effectiveness, achieving optimal retention remains a challenge because of the complexity of the tooth morphology. This study evaluated the impact of two viscosity-reducing methods (heat energy and sonic vibration) on flowability, adaptability, and long-term retention of flowable composite resin sealants.

Materials and methods: This quasi-experimental split-mouth study included 72 systemically healthy children aged 6-10 years. Teeth with shallow, wide fissures were allocated to the heat preheating group, whereas those with deep, narrow fissures were assigned to the sonic vibration group. The heat group utilized a composite resin heated to 50°C using an Endoking dental resin composite heater (Sigma Biomedicals, Telangana, India; power supply: DC 15 V, 2 A, 30 W), whereas the sonic group utilized a modified electric toothbrush (MI Xiaomi, MI electric toothbrush T100, Xiaomi Corp., Beijing, China) for sonic vibrations. The sealants were subjected to standardized etching, bonding, and photopolymerization protocols. Clinical evaluations were systematically performed at intervals of one, three, six, nine, and 12 months to evaluate the marginal integrity (MI), marginal discoloration (MD), and anatomical form (AF). Quantitative analysis was executed using the IBM SPSS Statistics for Windows, Version 23 (Released 2015; IBM Corp., Armonk, New York), with the threshold for statistical significance set at p<0.05.

Results: The investigation revealed 100% MI in both cohorts at one- and three-month intervals. At the six-month interval, the incidence of MI decreased in 69 (95.8%) cases in the sonic cohort and 61 (84.7%) in the heat cohort (p=0.024). Throughout the 12-month period, the sonic cohort consistently exhibited superior performance compared to the heat cohort in the preservation of MI and reduction in MD (p=0.020). While both cohorts demonstrated similar levels of AF of pit and fissure sealants at most time points, the sonic cohort displayed enhanced retention and a lower incidence of sealant loss.

Conclusion: Both the heat and sonic vibration methods effectively reduced the viscosity of sealants, improving their retention and adaptation. However, sonic vibrations demonstrated superior long-term performance, particularly for deep and narrow fissures.

## Introduction

Pit and fissure sealants have been effectively used to reduce the incidence of dental caries, particularly in the pediatric population [1]. Flowable composite resin sealants are frequently employed owing to their superior mechanical properties, which include significant wear resistance and strong adhesion to the enamel surfaces. However, achieving optimal retention of these sealants remains problematic due to the inherent complexities associated with tooth morphology [2]. Various strategies have been employed to enhance the bond strength and retention of pit and fissure sealants, including modifications of material characteristics, surface treatments, and application methodologies [1].

To enhance the flow characteristics in pits and fissures, it is crucial to reduce the viscosity of the material; however, this reduction may result in compromised mechanical properties. Consequently, the implementation of thermal energy and sonic vibrations during the application of flowable composite resins has recently garnered scholarly interest as a strategy to improve flowability, thereby enhancing material adaptation and retention [3]. The application of heat decreased the viscosity of the composite resin, facilitating superior penetration into deep pits, fissures, and micro-retentive regions. Furthermore, lowered viscosity can reduce the likelihood of voids and air entrapment, which are detrimental to the durability and structural integrity of the sealant [4].

Conversely, sonic vibration represents a dynamic methodology based on the principle of mechanical energy [5]. The vibrational forces enhance the fluidity of the resin and augment its capacity to infiltrate minute cavities within fissures. This leads to improved marginal integrity (MI) and diminished microleakage. Additionally, sonic vibration facilitates even distribution of the material, which further consolidates the adhesion to the enamel [6]. Research conducted by Kim et al. demonstrated that the efficacy of sonic vibrations surpassed that of heat applications [3].

Notwithstanding these promising theoretical advantages, there is a paucity of empirical evidence concerning the influence of thermal energy and sonic vibration on the durability of flowable composite resin sealants, particularly with extended follow-up outcomes. A quasi-experimental split-mouth study design provides a rigorous framework for assessing these variables as it facilitates a direct intraoral comparison of methodologies under standardized conditions. Consequently, this study sought to examine the impact of heat energy and sonic vibration on the durability of flowable composite resins utilized as pit and fissure sealants in pediatric populations.

## Materials and methods

Study design and setting

This prospective cohort quasi-experimental study was conducted in the Department of Pedodontics, Teerthanker Mahaveer Dental College and Research Centre, Moradabad, India, from July 2021 to December 2023. Ethical clearance was obtained prior to the beginning of this research (TMDCRC/IEC/2021/PPD2), and informed consent written in detail was signed by each patient's parents/guardians who were willing to participate in this study.

Sample size estimation

Sample size analysis was performed using the G*Power software version 3.6.9 (Heinrich-Heine-Universität Düsseldorf, Germany). A total of 122 samples were sufficient for the present study to determine significant changes in heat and sonic methods on sealant efficiency at 80% statistical power and a 5% alpha error, considering a minimum effect size of 0.45 [3]. Taking into account the dropout rate of 10%, 144 samples were selected. As the study followed a split-mouth quasi-experimental design, a total of 72 patients were estimated, contributing 144 teeth.

Eligibility criteria

This study included 72 healthy children aged 6-10 with fully erupted permanent mandibular molars having deep and narrow or wide and shallow pits and fissures on both sides, those who provided written consent, and cooperative children. Children with decayed, restored, developmentally defective permanent mandibular first molars, children with a high caries index, and children with poor oral hygiene were excluded.

Methodology

This study employed a quasi-experimental split-mouth design with fissure morphology-based allocation for 12 months. Teeth with shallow and wide fissures were assigned to the heat group, whereas teeth with deep and narrow fissures were allocated to the sonic vibration group. This design allows for the evaluation of technique-specific performance based on fissure depth and morphology, thereby providing a controlled comparison between the two application methods. In Group 1, the pit and fissure sealant heating was performed at 50°C (heat group), whereas in Group 2, the flowable composite resin was sonicated (sonic vibration group).

**Figure 1:**
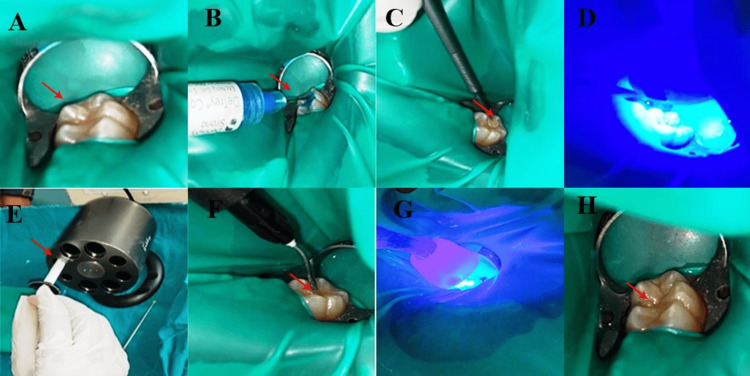
Sealant application on the occlusal fissure of a mandibular first molar tooth in Group 1: (A) rubber dam application, (B) etching with 37% phosphoric acid, (C) bonding agent application, (D) photopolymerization, (E) heating of composite, (F) composite filling, (G) photopolymerization, and (H) occlusal fissure filled with sealant.

The teeth selected for examination were the first permanent mandibular molars from both the left and right quadrants, which were initially isolated using a rubber dam to prevent salivary contamination (Figure 1A). Prior to the application of the sealant, the surfaces (pits and fissures) of the isolated teeth were cleaned to eliminate biofilm using pumice powder combined with distilled water. The etching procedure was performed using 37% phosphoric acid gel (D-Tech XT Etch, Pune, India) for 15 seconds (Figure 1B), followed by rinsing with distilled water and air-drying using oil-free compressed air for a period of 20 seconds. In Group 1, a bonding agent (Prime and Bond Universal, Dentsply Sirona, Germany) maintained at 50°C for five minutes was applied to the etched surface using an applicator tip, followed by photopolymerization (Figures 1C, 1D). Subsequently, flowable composite resin (A2 shade, Neo Spectra TM ST Flow, Dentsply Sirona, Germany), which was also kept at 50°C for five minutes (Figure 1E), was applied to the fissures, followed by photopolymerization as directed by the manufacturer for 20 seconds (Figures 1G, 1H). The heating device used in the heat group procedure was an Endoking dental resin composite heater (Sigma Biomedicals, Telangana, India; power supply: DC 15 V, 2 A, 30 W).

**Figure 2:**
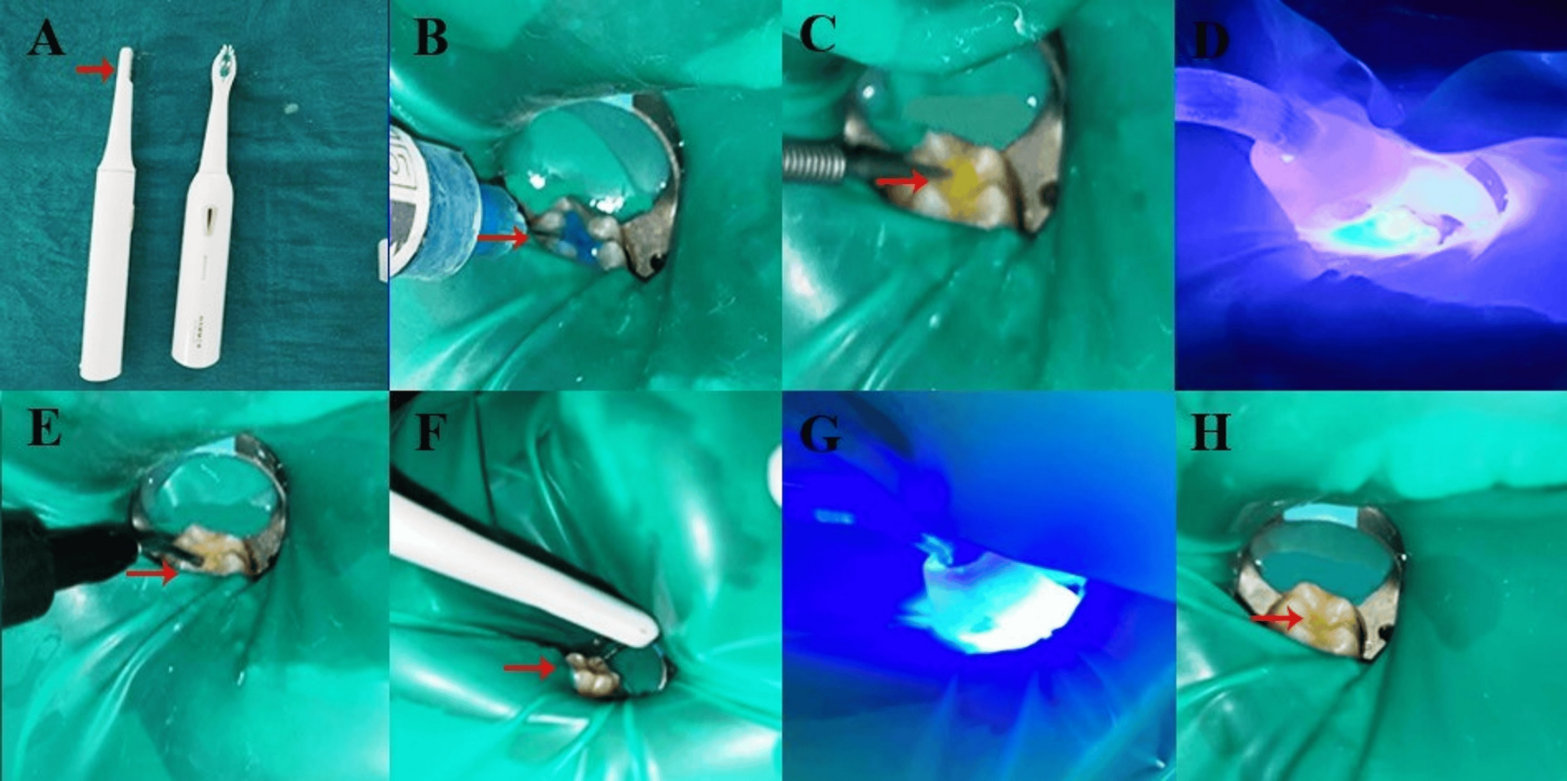
Sealant application on the occlusal fissure of a mandibular first molar tooth in Group 2: (A) modified toothbrush for sonic vibration, (B) etching with 37% phosphoric acid, (C) bonding agent application, (D) photopolymerization, (E) composite filling, (F) sonic vibration application, (G) photopolymerization, and (H) occlusal fissure filled with sealant.

In the sonic vibration group, a modified electric toothbrush (MI Xiaomi, MI Electric Toothbrush T100, Xiaomi Corp., Beijing, China) containing 0.5 mm from the handle of an electric toothbrush to the diameter spherical tip of a periodontal probe (Probe Bpcp10, Osung Mnd Company LTD., Gimpo, Korea) was used (Figure 2A). After etching (Figure 2B), the application of a bonding agent (Figure 2C), followed by photopolymerization (Figure 2D), an adhesive was used on the etched portion using an applicator tip (Figure 2E), followed by the application of sonic vibration using a modified toothbrush vibrating it horizontally and perpendicularly (Figure 2F), followed by photopolymerization (Figures 2G, 2H).

**Figure 3:**
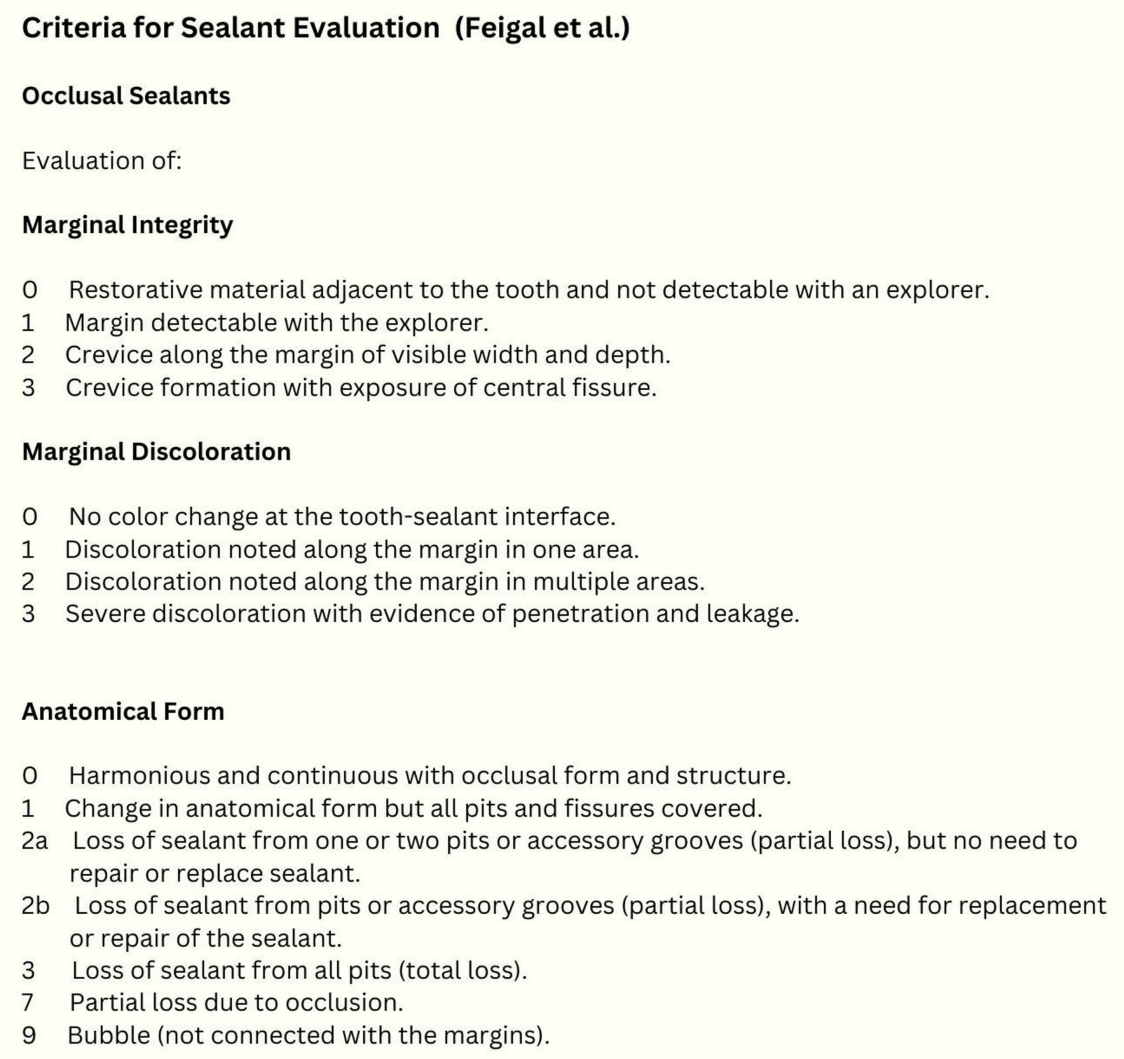
Criteria for sealant rating evaluation as outlined by Feigal et al. Reference: [7]

The modification was carried out in an electric toothbrush by removing the bristles from the brush head, and a periodontal tip was embedded into the brush head. The probe tip was secured to the brush head by heating it prior to securing it. The flowable composite resin, which was kept at room temperature, was applied to the fissures and sonicated using this modified electric toothbrush with a periodontal probe tip attached as described previously, followed by photopolymerization as directed by the manufacturer for 20 seconds. To evaluate the efficacy of both treatment modalities, clinical follow-up was performed on all teeth in both groups at one, three, six, nine, and 12 months. The success criteria were based on the criteria described by Feigal et al. (Figure 3) [7].

Statistical analysis

Descriptive statistics were calculated for the variables, with mean and standard deviation used to summarize quantitative variables and frequencies and proportions used for qualitative variables. The normality of the quantitative data was assessed using the Shapiro-Wilk test. Inferential statistical analyses included the independent t-test, chi-square test, and Fisher's exact test for qualitative variables. A significance level of 5% (p<0.05) was considered statistically significant. The analysis was conducted using the IBM SPSS Statistics for Windows, Version 23 (Released 2015; IBM Corp., Armonk, New York).

## Results

The study included 72 subjects with a mean age of 8.86 ± 1.27 years. Of these, 42 (58.33%) were males, and 30 (41.67%) were females. The comparison of mean ages between the sexes revealed no statistically significant difference (p=0.818). This indicates a comparable age distribution between the male and female participants in the study (Table 1).

**Table 1:** Mean age and sex distribution for the study subjects. *Independent t-test; p-value>0.05: non-significant. The number of males and females is represented as n (%), and their age is represented as mean and standard deviation (SD).

Sex	N (%)	Mean (Years)	SD	t-statistic	p-value
Male	42 (58.33%)	8.83	1.27	0.230*	0.818
Female	30 (41.67%)	8.90	1.27		

A 100% MI was maintained in both groups at the one- and three-month follow-up visits. At the six-month interval, the MI remained high in 69 (95.8%) children for Group 2, whereas it was seen in 61 (84.7%) for Group 1, with a statistically significant difference (p=0.024). At nine months and 12 months, the MI decreased in both groups with non-significant differences; however, the scores were better in Group 2 than in Group 1 (p>0.05). This showed that although both groups showed no statistically significant differences at most time intervals, the performance of the sonic group was better than that of the heat group at follow-up visits (Table 2).

**Table 2:** Clinical assessment of marginal integrity (MI) among different groups at different time intervals. *p-value<0.05: significant (S); p-value>0.05: non-significant (NS). Data are represented as n (%).

Time Interval	Score (MI)	Heat		Sonic		Chi-Square Test		
		N	%	N	%	Chi-statistics	p-value	Significance
1 month	0	72	100.0%	72	100.0%	NA	NA	NA
3 months	0	72	100.0%	72	100.0%	NA	NA	NA
6 months	0	61	84.7%	69	95.8%	5.06	0.024*	S
	1	11	15.3%	3	4.2%			
9 months	0	58	80.6%	67	93.1%	5.23	0.072	NS
	1	9	12.5%	4	5.6%			
	2	5	6.9%	1	1.4%			
12 months	0	55	76.4%	65	90.3%	5.51	0.063	NS
	1	12	16.7%	6	8.3%			
	2	5	6.9%	1	1.4%			

At the one- and three-month intervals, no marginal discoloration (MD) was observed in any of the patients in either group. At the six-month interval, MD was observed in seven (9.7%) cases in Group 1 and in two (2.8%) cases in Group 2, with no statistically significant difference (p>0.05). At the nine-month interval, the MD scores decreased in both groups, showing signs of discoloration, which was higher in Group 1, with no statistically significant difference (p>0.05). At 12 months, statistically significant discoloration was observed in Group 1 compared to that in Group 2 (p=0.020), as shown in Table 3.

**Table 3:** Clinical assessment of marginal discoloration (MD) among different groups at different time intervals. *p-value<0.05: significant (S); p-value>0.05: non-significant (NS). Data are represented as n (%).

Time Interval	Score (MD)	Heat		Sonic		Chi-Square Test		
		N	%	N	%	Chi-statistic	p-value	Significance
1 month	0	72	100.0%	72	100.0%	NA	NA	NA
3 months	0	72	100.0%	72	100.0%	NA	NA	NA
6 months	0	65	90.3%	70	97.2%	2.96	0.085	NS
	1	7	9.7%	2	2.8%			
9 months	0	62	86.1%	68	94.4%	2.84	0.091	NS
	1	10	13.9%	4	5.6%			
12 months	0	55	76.4%	67	93.1%	7.74	0.020*	S
	1	13	18.1%	4	5.6%			
	2	4	5.6%	1	1.4%			

The clinical assessment of the anatomical form (AF) showed no statistically significant difference between the groups at any time point. At the one- and three-month intervals, AF was maintained in 72 (100%) cases in both groups. At the six-month interval, a change in AF was observed in seven (9.7%) cases in Group 1 and two (2.8%) cases in Group 2. At nine months, a change in AF was observed in a greater number of cases in both groups. At the 12-month interval, the total loss of sealant from the pits was observed in four (5.6%) cases in Group 1 and one (1.4%) case in Group 2 (Table 4).

**Table 4:** Clinical assessment of anatomical form (AF) among different groups at different time intervals. A p-value of >0.05: non-significant (NS). Data are represented as n (%).

Time Interval	Score (AF)	Heat		Sonic		Chi-Square Test		
		N	%	N	%	Chi-statistic	p-value	Significance
1 month	0	72	100.0%	72	100.0%	NA	NA	NA
3 months	0	72	100.0%	72	100.0%	NA	NA	NA
6 months	0	68	94.4%	70	97.2%	0.69	0.40	NS
	1	4	5.6%	2	2.8%			
9 months	0	63	87.5%	69	95.8%	3.27	0.07	NS
	1	9	12.5%	3	4.2%			
12 months	0	61	84.7%	67	93.1%	2.11	0.44	NS
	1	9	12.5%	4	5.6%			
	2	1	1.4%	1	1.4%			
	3	1	1.4%	0	0.0%			

## Discussion

The increased viscosity of the pit and fissure sealants adversely impacts the adaptability of the sealant to the dental substrate and hinders thorough penetration, thereby diminishing the retention of the sealant in the tooth structure. Conversely, sealants with low viscosity demonstrated an enhanced capacity for the distribution and rapid infiltration of the tooth surface. The extent of penetration is a crucial determinant that can enhance the longevity, resilience, and compatibility of fissure sealants. The results of the present study indicated that although both groups were effective in reducing viscosity and thus maintaining MI, AF, and preventing MD, the sonic group performed better than the heat group at more than a six-month follow-up.

Sonic vibrations led to shear stresses and increased the fluidity of the flowable composite without affecting its mechanical properties. Similar results have been reported by Kim et al. [3]. The application of vibrational treatment induces the transition of thixotropic substances into a liquid-like condition; upon the cessation of vibration, these materials revert to their initial solid-like form. This thixotropic characteristic modifies viscosity and enhances flow attributes [8]. The flowable composite resins exhibited a reduction in viscosity and displayed a thixotropic behavior as the shear rate increased. This implies that flowable resin composites possess an enhanced capability to flow autonomously within narrow cavities and fissures as the shear rate increases [9]. The application of shear force to resin composites demonstrates inadequate molecular cohesion, and the rearrangement of filler particles results in the degradation and compromise of the molecular bonds. Consequently, this process induces a decrease in viscosity, thereby enhancing the ability of resin composites to flow more freely [10].

Kersten et al. noted that the application of ultrasonic vibration during the etching process of dental structures enhanced the efficacy of fissure sealing, facilitating deeper infiltration of the acid gel into the enamel matrix [11]. The preheating of composite materials improves the degree of direct contact and marginal adaptation [12], which may be attributed to the reduced viscosity of the substance resulting from the heating procedure. This process can enhance the kinetic activity of molecules, facilitating superior cross-linking and increasing efficacy [13].

In the current study, the Endoking dental resin composite heater was utilized to produce heat energy based on the assumption that a chairside preheating protocol could be implemented. Traditionally, composite restorations have been subjected to preheating using composite warming trays, water baths, or composite heaters [14]. These techniques are characterized by their susceptibility to procedural variability and substantial temporal requirements. Moreover, the effectiveness of preheating is a matter of debate because of the rate at which the ambient temperature is lost. It has been reported that the temperature decreases by 50% within a mere two minutes following the removal of a composite from a heating device [15]. As a result, preheating performed away from the chairside during clinical procedures is unlikely to produce the anticipated results. In this study, preheating of the composite material was carried out within the dental cavity, culminating in a significant rise in temperature, which has the capacity to enhance the thermal conditions within the cavity. Therefore, this study used this method for shallow and wide fissures.

Clinical implications

This investigation underscores the premise that the application of sonic vibrations markedly improves the adaptability and penetration of fissure sealants by reducing viscosity, resulting in enhanced retention and longevity. The thixotropic characteristics inherent in flowable composites activated by shear stresses facilitate deeper infiltration into constricted fissures and cavities, thereby mitigating the potential for microleakage and carious lesions. In contrast to the methodology of heat preheating, the utilization of sonic vibrations has demonstrated superiority in preserving flowability while maintaining mechanical properties, particularly over a follow-up period of six months. It is imperative for clinicians to consider the implementation of chairside sonic vibration devices for fissure sealant procedures to optimize clinical outcomes, augment marginal adaptation, and improve the comprehensive efficacy of both preventive and restorative interventions. Our study also recommends using a fluoride-releasing pit and fissure sealants for additional anti-cariogenic effects.

Limitations

The clinical efficacy of fillers is influenced by their composition, categorization, and morphological characteristics. Consequently, future in vitro and clinical investigations are essential for assessing these factors. Additionally, a further limitation of this research is the necessity to analyze various categories of flowable, bulk, nanohybrid, and microhybrid commercial composites to achieve more comprehensive comparative findings. Prior studies have assessed the marginal adaptation and water sorption properties following the application of preheated composites [16]. Therefore, future studies should focus on interfacial bonding, mechanical attributes, such as flexural and compressive strengths, and water sorption properties.

## Conclusions

The study results demonstrate that both sonic vibration and heat application effectively reduce the viscosity of pit and fissure sealants, improving their marginal adaptation and retention. However, no significant differences were observed in the anatomic form of the sealants. The sonic group showed superior MI at the six-month follow-up and lower MD at the 12-month follow-up compared to the heat group. Consequently, the sonic vibration method proves to be a more effective choice, particularly for deep and narrow fissures, ensuring better long-term performance and retention of sealants in such cases.
